# Point-of-care ultrasound detection of tracheal edema caused by smoke inhalation

**DOI:** 10.1186/2036-7902-6-S1-A26

**Published:** 2014-01-31

**Authors:** T Kameda, M Fujita

**Affiliations:** 1 Department of Emergency Medicine, Red Cross Society Azumino Hospital, Azumino, Japan

## Background

Smoke inhalation is the leading cause of death due to fires. When a patient presents with smoke inhalation, prompt assessment of the airway and breathing is necessary. Point-of-care ultrasound is used for the rapid assessment of critically-ill or injured patients. To the best of our knowledge, the use of point-of-care ultrasound for the detection of tracheal edema caused by smoke inhalation has never been reported in the English literature.

## Case presentation

A 54-year-old male was transferred to the emergency department with an increasingly evident cough, carbonaceous sputa and rhinorrhea about six hours after inhaling smoke caused by a fire in his locked bedroom. On examination, he was alert. His oxygen saturation was 94% on 2 liters of oxygen by nasal cannula, with a respiratory rate of 25 breaths/min, and his heart rate was 106 beats/min, his blood pressure was 151/100 mmHg and his body temperature was 37.3°C. He had no surface burns on the face and no edema or erosion in the oral cavity. He had hoarseness without stridor. His breath sounds were positive for expiratory wheezes. Chest X-rays indicated narrowing of the trachea. Laryngoscopy showed light edema and erosive findings on the supraglottic region. Bedside point-of-care ultrasound revealed hypoechoic thickening of the tracheal wall, which was consistent with tracheal edema. The thickening was confirmed by a computed tomographic scan. The patient was carefully monitored with preparation for emergency airway management and was treated with supplemental oxygen, an aerosolized β2 adrenergic agonist and a single intravenous administration of methylprednisolone. The symptoms were subsequently relieved, and reexamination by ultrasound after two days showed remission of the wall thickening.

## Conclusion

Point-of-care ultrasound may be a useful modality for the quick diagnosis and follow-up of tracheal edema caused by smoke inhalation.

